# Diverse nanocomposites as a potential dressing for diabetic wound healing

**DOI:** 10.3389/fendo.2022.1074568

**Published:** 2023-01-11

**Authors:** Remya Rajan Renuka, Angeline Julius, Suman Thodhal Yoganandham, Dhamodharan Umapathy, Ramya Ramadoss, Antony V. Samrot, Danis D. Vijay

**Affiliations:** ^1^ Centre for Materials Engineering and Regenerative Medicine, Bharath Institute of Higher Education and Research, Chennai, Tamilnadu, India; ^2^ Department of Environmental Engineering, Institute of Industrial Technology Changwon National University, Changwon, Gyeongsangnamdo, Republic of Korea; ^3^ School of Smart and Green Engineering, Changwon National University, Changwon, Gyeongsangnamdo, Republic of Korea; ^4^ Department of Research, Karpaga Vinayaga Institute of Medical Science and Research Centre, Madhuranthagam, Tamilnadu, India; ^5^ Department of Oral Biology, Saveetha Dental College, Chennai, Tamilnadu, India; ^6^ School of Bioscience, Faculty of Medicine, Bioscience and Nursing, MAHSA University, Jenjarom, Selangor, Malaysia

**Keywords:** nanocomposites, scaffolds, hydrogen-based scaffolds, Chitosan-based scaffolds, diabetic wound healing

## Abstract

Wound healing is a programmed process of continuous events which is impaired in the case of diabetic patients. This impaired process of healing in diabetics leads to amputation, longer hospitalisation, immobilisation, low self-esteem, and mortality in some patients. This problem has paved the way for several innovative strategies like the use of nanotechnology for the treatment of wounds in diabetic patients. The use of biomaterials, nanomaterials have advanced approaches in tissue engineering by designing multi-functional nanocomposite scaffolds. Stimuli-responsive scaffolds that interact with the wound microenvironment and controlled release of bioactive molecules have helped in overcoming barriers in healing. The use of different types of nanocomposite scaffolds for faster healing of diabetic wounds is constantly being studied. Nanocomposites have helped in addressing specific issues with respect to healing and improving angiogenesis. Method: A literature search was followed to retrieve the articles on strategies for wound healing in diabetes across several databases like PubMed, EMBASE, Scopus and Cochrane database. The search was performed in May 2022 by two researchers independently. They keywords used were “diabetic wounds, nanotechnology, nanocomposites, nanoparticles, chronic diabetic wounds, diabetic foot ulcer, hydrogel”. Exclusion criteria included insulin resistance, burn wound, dressing material.

## Introduction

**Figure 1:**
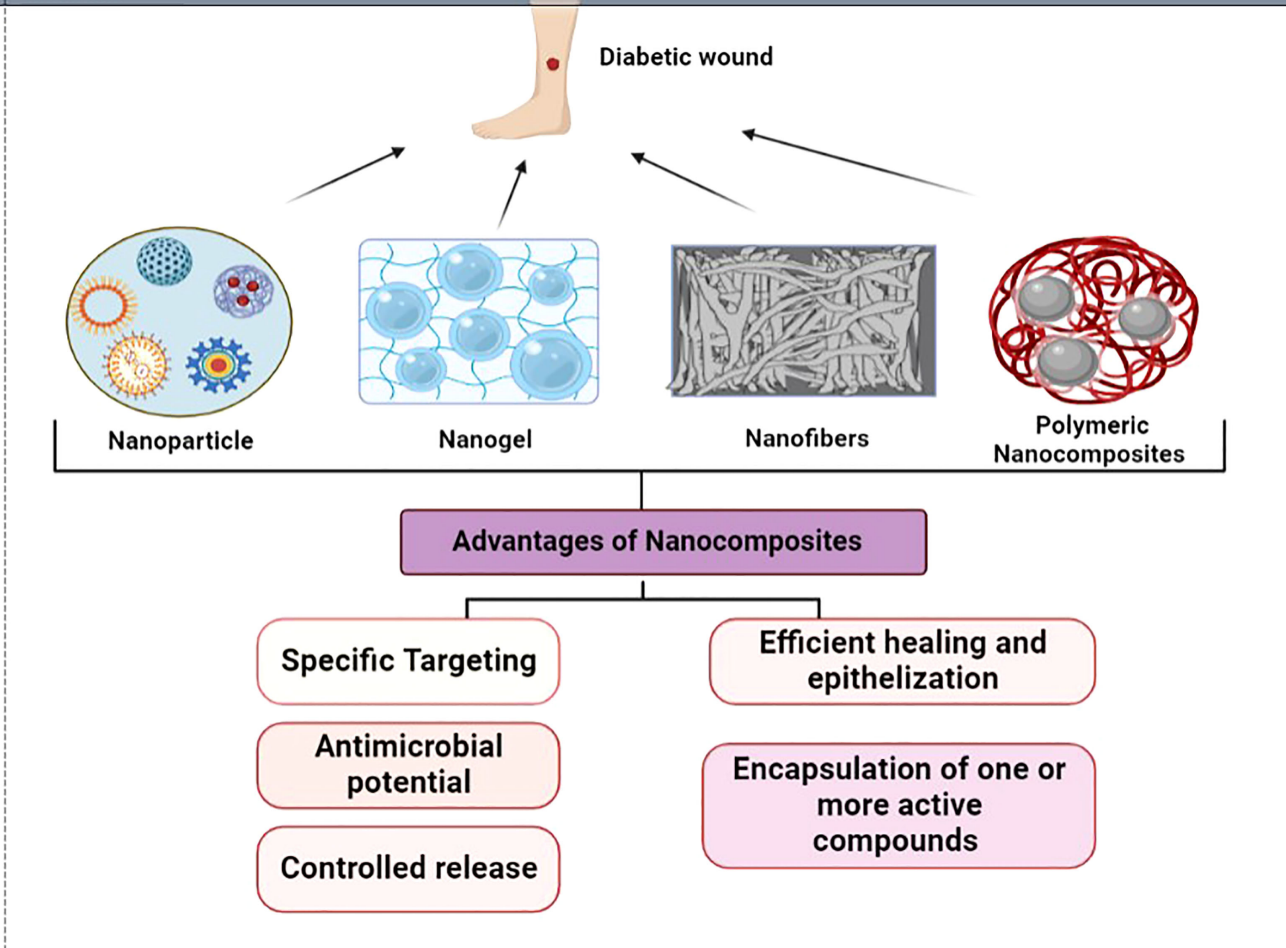
Types of nanocomposites and its advantages as a wound dressing.

Nanomedicine is one of the fastest-growing fields offering several avenues for therapy, diagnostics, delivery systems and improving efficiency (1). The superior properties of the ‘nano’ components have been used in tissue engineering for the repair and regeneration of several organs and tissues. Wound healing is a normal process involving a series of steps; however it is affected by a number of variables like age, obesity, stress, diseases, habits, infections, trauma etc. (2). But in certain conditions, healing is halted at the second phase, i.e., the inflammatory phase which could be due to chronic conditions. Many diseases that cause impaired blood flow, such as in the case of diabetic foot ulcers or pressure ulcers are the contributing factors. Common wound pathogens, nosocomial infections are also known to hinder the progression of healing to the third phase, which is proliferation. Several factors are attributed to patients with diabetes mellitus such as the improper function of macrophages and growth factors, and low blood circulation; the major factor for delayed wound healing (3). The incidence of diabetes is seen to be increasing at a steady rate globally, with a mortality rate of 1.5 million deaths in 2019. The indirect death due to diabetes was 460,000 due to kidney disease, and 20% due to cardiovascular complications (4). Diabetic patients are prone to develop diabetic foot ulcers and the percentage affected is more than 20% (5). To treat such chronic wounds, newer therapies such as cell/gene therapy, and engineered biomaterials are sought after due to unsuccessful treatment modalities. Tissue engineering has led researchers to explore several new skin substitutes using natural, synthetic, and semi-synthetic polymers. They are often used in combination with biomolecules, proteins, and polysaccharides (6). To overcome the existing limitations, they have been combined with nanomaterial to form a highly functional, multi-modal, smart nanocomposite to treat chronic wounds such as in the case of diabetes (7). The major advantages of nanotherpay are due to the charge, and large surface area to volume ratio that enhances the interaction with the target area (8). The ability to encapsulate and control the drug release by attaining a sustained release of the desired biomolecules leads to accelerated healing (9). **Figure 1** represents the types of nanocomposites and its advantages in wound healing.

Various factors, including pH, temperature, blood sugar level, and oxygen saturation, are important in the healing of wounds. Scaffolds have attracted interest recently as a novel dressing and provide an innovative perspective on tissue regeneration (10, 11). Researchers state that the design of the dressing material spatially is of utmost importance for a biomaterial to function as an effective regenerative scaffold, which is now possible through nanocomposites which have been summarised in this review.

## Nanoparticle based composite scaffold for enhanced healing

While researchers are experimenting with effective and scarless wound healing strategies, wound management in diabetes itself is a tedious process for the patients and the healthcare sector. So, several advanced techniques and technology have been employed in the remedial measures for diabetic wounds.

Several types of metal nanoparticles, metal oxide nanoparticles, nanotubes, and polymeric nanoparticles have been used in wound healing. Because of their innate antimicrobial property, the silver nanoparticle has been extensively used in wound care management. It is strikingly important to note that silver nanoparticles are effective against nosocomial infections and multidrug-resistant pathogens (12, 13). At a concentration of 50 mg/ml silver nanoparticles were observed to destruct the bacterial cell membrane and kill *S. aureus* and *E. coli* (14).

A nanocomposite was fabricated with polyethylene glycol diacrylate, silica, bioactive glass nanoparticles, sodium alginate and copper. This silica-based nanocomposite was found to be an excellent injectable with elastomeric, biomimetic, and antibacterial properties. The regeneration of blood vessels was observed with high collagen deposition, and VEGF expression in a full-thickness diabetic wound model (15). **Table 1** indicates the different types of nanocomposites employed for diabetic wound healing.

**Table 1:** Types of nanocomposites employed for diabetic wound healing.

Nanocomposite	Role in wound healing	Reference
Polyurethane nanoparticles	Induces angiogenesis, cell proliferation	(16)
AuNPs with epigallocatechin and lipoic acid	Regulated angiogenesis and inflammation to accelerate faster healing	(17)
Bioactive glass with Cu	Stimulation of CD31, HIF-1α, VEGF expression. Antibacterial activity	(18)
Silicate Bioglass NPs	Increased proliferation of epithelial cells and nitric oxide expression that enhanced angiogenesis	(19)
45S5 bioglass with Strontium and Copper	Aided the differentiation of stem cells to vascular endothelial cells, formation of tubular vein endothelial cells.	(20)
CuNPs in carbon nanofibers	Upregulation of placental growth factor, VEGF, hypoxia inducible growth factor. Increased vascularisation and wound closure rate	(21)
CuNPs in hyaluronic acid hydrogel	Upregulation of the growth factor, VEGF. Promoted angiogenesis and collagen deposition	(22)
PCL nanofibers with curcumin	Distinct granulation tissue formation. Increased fibroblast proliferation, collagen content, and faster regeneration.	(23)
AgNPs in hyaluronic acid nanofibers	Antibacterial activity. Accelerated healing in wounds	(24)
Cellulose nanocrystals in PLGA fibers	Inflammatory cytokines, IL-1 and IL-6 were reduced. Higher rate of epidermal and dermal regeneration.	(25)
Chitosan in PVA nanofibers	Upregulation of HIF-1 and VEGF. Improved interaction among endothelial cells and fibroblasts	(26)

Nitric oxide (NO) induces the formation of blood vessels and the migration of endothelial cells by eNOS or MAPk pathways. Zinc oxide is known to induce NO production, hence several scaffolds for wound healing have incorporated ZnO NP’s nanofibers fabricated using poly-caprolactone with ZnO NP’s which exhibited high proliferation of fibroblast cells. A higher rate of vascular regeneration was observed because of the expression of VEGF and FGF (27). Cerium oxide nanoparticles were used in combination with microRNA (miR-146a) for faster healing in diabetic wounds. The synergistic role of scavenging the free radicals and modulating the inflammatory pathway proved to increase the synthesis of collagen, thereby higher rate of angiogenesis and low inflammation; this aided in a significantly higher rate of wound closure (28).

Poly-N-acetyl-glucosamine based nanofibrous scaffold was prepared to overcome the limitations in treating a diabetic wound. This bioactive scaffold was found to enhance cell metabolism, and migration of endothelial cells with a higher rate of wound closure in a full- thickness diabetic mice model. The gene expression of uPAR, VEGF, Il-1 and MMP responsible for migration, angiogenesis, inflammatory activity, and matrix remodelling was observed (29). Another study found that short-fibre poly-N-acetyl glucosamine nanofibers were used alongside the vacuum-assisted closure of complex wounds. This aided in controlling the blood loss by acting as a hemostatic agent activating platelets and better granulation. The presence of collagen I and the wound contraction rate was significant in the treated groups (30).

## Stimuli-responsive scaffold for modulated healing process

The ulcers in diabetic wounds are caused by oxidative stress, so researchers prepared Prussian blue nanoparticles (PBNP’s) to scavenge the free radicals generated at the wound site. This PBNP was encapsulated in a heat-sensitive gel using poly (d, L-lactide)-poly (ethylene glycol)-poly (d, L-lactide) (PDLLA-PEG-PDLLA) hydrogel (PLEL). It was confirmed that the nanoparticle was able to protect the cells and mitochondria against reactive oxygen species (ROS). In an animal model, it was found to progress diabetic wound healing at a faster rate, reduce ROS production, and enhance cell survival and growth simultaneously reducing the interleukin and tumor necrosis factor (31).

A pH-responsive scaffold was developed which aided in faster healing with less scar formation. This injectable scaffold was prepared with polysaccharides and exhibited antibacterial activity against multi-drug resistant bacteria. *In vivo* studies showed that the exosome released promoted angiogenesis in the full-thickness wound (32). A dual responsive scaffold that modulates the release based on pH and metformin release was prepared using PEG. The active components encapsulated were phenylboronic acid, benzaldehyde, L-arginine, and chitosan which exhibited anti-inflammatory effects and promoted angiogenesis. The synergistic healing of metformin and graphene oxide was observed in a rat model with type II diabetic foot ulcer. Based on the stimulus it was found to release the drug, metformin which was faster healing in chronic diabetic athletic wounds (33).

Silver nanoclusters were conjugated with vancomycin in a gelatin-based hydrogel along with nimesulide that is pH sensitive. This complete biomaterial containing phenylboronic acid and polyvinyl alcohol also contained ROS and exhibited anti-inflammatory action. It was found to be biocompatible, with excellent cell-adhesive behaviour and aided healing in wounds with infection. Because of its sensitive and dual-responsive properties, hydrogel was found to be good for treating chronic diabetic wounds (34). A thermos-responsive scaffold that is skin-friendly and designed for infants and diabetic patients with sensitive skin was attempted by researchers. This non-irritable hydrogel patch was designed with a protein-polyphenol complex that was activated upon reaction to the body temperature upon application. This was found to be skin-friendly and gentle even for a prolonged period of use because of its immune-modulatory action (35).

## Hydrogel-based scaffold

Hydrogels are the most preferred dressing agent for wound healing owing to their capability to retain moisture at the site of wounds, agent because hydrogels are designed to hold moisture at the wound surface, and create the best setting for healing, balancing skin hydration and in the removal of necrotic tissue. They could be prepared with ease providing sustained drug release. Both natural and synthetic polymers could be used in the preparation of hydrogels. These may include, fibrin, hyaluronic acid, cellulose derivatives, copolymers and others (36).

Hydrogels are exceptional in providing a humid atmosphere for the healing of wounds and ensure permeable water vapours with microbial entry prevention at the wound site. A heparinised PVA-based hydrogel formulated demonstrated significant antibacterial activity without any cellular toxic effects (37). Another hydrogel containing coumestrol/hydroxypropyl-β-cyclodextrin was developed using hydroxypropyl methylcellulose. The insoluble coumestrol (helps with photoaging; improves elasticity of skin during menopause) was solubilized using hydroxypropyl-β-cyclodextrin to obtain a hydrogel which led to faster wound healing process through the better propagation of cells. This also demonstrated good cell adhesion and compatibility as observed through Wistar rats (38). A gel-based hydrogel was formulated using adipose-derived stem cells as a suitable wound healing agent that was obtained from both mouse and porcine models. These *in vivo* models demonstrated excellent healing of wounds (39). Topic nitric oxide helps in the healing process of acute and chronic wounds. An antibacterial peptide was developed based on this, which could self-assemble with respect to changes in pH, and could lead to the development of hydrogel with improved bactericidal activity (40). A ZnO-based nanocomposite hydrogel demonstrated significant antibacterial properties and was found biocompatible and safe with a faster rate of wound healing (41). Though there are many ongoing research on hydrogels related to skin repair, there is another group of researchers who developed hydrogels containing HA and carboxylated CS that mimics skin with high mechanical strength. The *in vitro* studies on L929 cells demonstrate superior biocompatibility with improved cell proliferation. Further, *in vivo* studies also demonstrated a faster healing process and suggested that this hydrogel as an ideal candidate suited for wound recovery and healing (42). Though there are many ongoing research on hydrogels used as wound dressing agent, we would like to identify the importance of hydrogels as a potential wound dressing agent with reference to diabetic wounds.

Diabetes being a chronic disease is yet challenging to cure and the medical requirements are inadequate (43). The skin wounds caused by diabetes do not get completely healed due to limited blood supply and deprived antimicrobial capability with the poor inflammatory response (44). Among 750,000 emerging cases of diabetic foot ulcer in America, nearly 10% of cases involved amputation of limbs every year (45). Many measures are taken for treating wound healing due to diabetes such as growth factor and cellular-based therapy but the cost was too high (46–48). So, there has been an increasing interest in bioactive biomaterials as a potent wound dressing agent for treating in case of diabetic-based wounds (49). Some of the biomaterials have progressed to clinics such as biomedical hydrogels, films and ointments, and others (50). On the other hand, multifunctional biomaterials are developed with potent antioxidants, antibacterial activity and hemostasis (51–53). Hydrogel biomaterial-based dressings are also developed with their property similar to that of the extracellular matrix and this demonstrated good wound healing (54–58).

Due to vascular impairment, diabetes-related wound healing and skin regrowth remain a major concern. To overcome this, a silica-based nanocomposite hydrogel scaffold that could promote both wound healing and skin regeneration in diabetic conditions was developed by enhancing early angiogenesis with no bioactive factors. This injectable nanocomposite exhibits an excellent healing pattern with superior antibacterial properties. Also, enables viability, growth, and angiogenesis of endothelial progenitor cells through *in vitro* studies. *In vivo* studies demonstrated restoration of blood vessels through HIF-1α/VEGF and collagen deposition in diabetic wound. It was also suggested to have its application in regenerative medicine (15). Several tissue engineering strategies using nanobiomaterials for vascular regeneration have been reported (59). A multifunctional sprayable cross-linking bioadhesive hydrogel-based nanocomposite was developed for diabetic wound healing. Here, Kappa-carrageenan being the hydrogel matrix, different concentrations of modified ZnO nanoparticles were incorporated to improve their mechanical properties with good antibacterial activity. To this, L-glutamic acid was also loaded into this network to enhance the rate of wound healing. This biocompatible nanocomposite also demonstrated elasticity similar to human skin with adhesive nature and clotting capability. The *in vivo* studies further demonstrated significant wound healing at a faster rate without any infection (59). A 2-D nanoclay (Laponite RD)/polymer-based nanocomposite hydrogels were developed as a substitute for treating foot ulcers due to diabetes. It was also suggested that enzymes or active compounds loaded to the hydrogel could help in the healing of diabetic foot ulcers through their antibacterial activity (60). Another research on zwitterionic poly (sulfobetaine acrylamide) nanocomposite that was composed of hectorite nanoclay demonstrated as a potent chronic wound dressing agent. This hydrogel exhibited insignificant cytotoxicity against NIH-3T3 fibroblast and was resistant against the adsorption of BSA and certain bacterial strains. *In vivo* studies on both normal and diabetic wounds were conducted in mice in comparison with commercially available dressings. Histology confirmed significant re-epithelialization and faster healing of diabetic wounds than the commercial products (61). A bioactive HQB nanocomposite hydrogel was developed through the cross-linking of modified hyaluronic acid with quaternized chitosan coated with bioactive glasses. This demonstrated superior wound healing properties in diabetic-induced rats and suggested it to have a good prospect in clinical application (62). A cost-effective and simple dual-network hydrogel comprised of MnO2 nanosheets was developed from silk fibroin and carboxymethyl cellulose. This helped in angiogenesis, reduced inflammation, and had remarkable healing rates comparable to commercial dressing through *in vivo* studies (63). An alginate and Eudragit nanoparticle-based nanocomposite hydrogel comprising edaravone was produced for the highest ROS sequestration to overcome chronic inflammation and delayed wound healing in diabetes. A lower dosage of this hydrogel enhanced wound healing, and a higher dosage impeded the healing process in diabetic mice and suggested dosage levels played a key role in the healing process (64). Some examples of nanoparticle-based wound dressing materials for which clinical trials are undertaken is listed in **Table 2**.

**Table 2:** List nanocomposite scaffolds undergoing/completed clinical trials.

Nanocomposite	Wound type	Reference
Hydrogel/Nano Silver-based Dressing	Diabetic foot wound	(65)
Wound Dressing FibDex (Nanofibrillar cellulose)	Dermal burn	(66)
AgNP	Partial thickness burns	(67)
Ag nylon	Surgical wound	(68)
AgNP- Acticoat	Fresh burn	(69)
Hydrofibre of Ag	Pilonidal sinus	(70)
Nanocrystalline silver	Leg ulcer	(71)

## Chitosan-based scaffolds

The major risk associated with patients affected with diabetes includes delayed wound healing and amputation. This is mainly due to the reduced tissue blood circulation causing hypoxia and the associated risks. A PVA/Chitosan-based nano fibre wound dressing was developed with high antimicrobial activity, improved vapour transmission rate, good odour-absorbing capacity and no cytotoxic effects; and was proved to accelerate the diabetic wound healing when tested in both diabetic and non-diabetic rats (72). A safe, cyto-compatible, epidermal growth factor-modified curcumin-incorporated chitosan nano-spray was developed that demonstrated accelerated wound healing properties, improved angiogenesis, and re-epithelialization with superior antibacterial effects in rats. It was further suggested that this nano-spray could help in the treatment of diabetic wounds and other skin injuries (73). A formulation composed of poly lactic acid/chitosan nanoscaffolds encapsulating cod liver oil was developed and characterized which demonstrated significant wound healing property to be used in the treatment of the most complicated disorder, diabetic foot ulcers, seen in diabetic patients (74). A topical formulation of lecithin-chitosan nanoparticles incorporated with melatonin was developed with desirable properties such as fibroblast induction, collagen deposition and promotion of angiogenesis. The formulation demonstrated4 accelerated wound closure in diabetic rats (75). A nanocomposite sponge comprised of chitosan, hyaluronic acid and nano-silver was developed against many antibiotic-resistant bacteria including methicillin-resistant *S. aureus*. The excellent antibacterial action exhibited by this nanocomposite sponge made it a suitable dressing agent for diabetic foot ulcers with mild toxicity towards mammalian cells (76). A hydrogel membrane composed of polyvinyl alcohol, starch, chitosan and nano zinc oxide was prepared and was found effective as a potent wound dressing agent in initial wound healing stages through *in vivo* studies in rats and exhibited wide-spectrum antibacterial action through *in vitro* studies (77). An injectable nanocomposite composed of curcumin, chitosan and alginate was identified as a promising wound dressing agent for wound recovery. The *in vivo* studies in rats demonstrated that the nano-curcumin based nanocomposite showed significant collagen deposition and epidermis re-epithelialization in wounds (78).

Although there is a gap in the translation of nanomedicine, the use of computer-aided analysis has become evident in this area. This has led researchers get a clear vision on the behaviour and application of nanoparticle- based therapy in reproductive biology (79), transporting drugs across biological barriers like the blood-brain barrier (80, 81). Another important aspect with respect to the design and use of nanotherapeutics is the toxicity; the understanding of which has been highly enhanced using computational biology (82). Several machine-learning approaches have been explored by the researchers that gives a magnified view of the interaction of the nanomaterial with the cells. This enables tailor-made and non-toxic application of nanomedicine to improve healthcare (83).

## Conclusion and future perspective

Several impeding factors in healing chronic wounds exist using conventional treatment methods. Novel strategies have been designed to overcome them using nanotechnology has proved to be promising. The advanced biomaterials developed with cellular and acellular scaffolds in conjunction with nanomaterials of suitable nature would prove to be an efficient wound care management in diabetic ulcers. The ability to modulate and control the release of active compounds and drugs has added advantage of controlling infection in these wounds significantly shortening the stay at the hospital for patients. An in-depth analysis of the factors that promote angiogenesis and wound closure at a faster rate by the use of nanocomposite biomaterials would help in translating these products to patients. Production of such tailor-made biomaterial constructs with specific factors and design would be the desired wound treatment strategy specifically in chronic wounds. With the recent advancements in the field of artificial intelligence the design of scaffolds could be customised to evade toxicity and meet the scrupulous needs that exist in regenerative therapy. This would help researchers validate and predict the outcome of their research without the sacrifice of many animals, avoid the strain of extraneous tasks involving the toxicological assessment. So, by means of integrating artificial intelligence and the lab-scale studies will yield effective translation of nanotherapeutics in wound care management.

## Author contributions

RRR and DV conceptualized the manuscript and performed the literature search and AJ and SY drafted the manuscript. RR, DU and AS reviewed and modified the manuscript. All authors contributed to the article and approved the submitted version.
